# The Roles of Parent-and-Child Mental Health and Parental Internet Addiction in Adolescent Internet Addiction: Does a Parent-and-Child Gender Match Matter?

**DOI:** 10.3389/fpubh.2020.00142

**Published:** 2020-05-15

**Authors:** Lawrence T. Lam

**Affiliations:** ^1^Tung Wah College, Hong Kong, China; ^2^Faculty of Health, The University of Technology Sydney, Sydney, NSW, Australia

**Keywords:** internet addiction, parental mental health, depression, stress, dyad study

## Abstract

**Objective:** This study aimed to investigate the relationship between parental mental health, particularly depression, and Internet addiction (IA) among adolescents taking into consideration adolescent mental health and parental IA as possible mediating factors. Of particular interest was the effect of parent-and-child gender match on these relationships.

**Materials and Methods:** This was a population-based parent-and-child dyad health survey utilizing a random sampling technique. Adolescent IA was measured by the Internet Addiction Test (IAT) designed by Young. The mental health status of the parents was assessed using the Depression, Anxiety, Stress Scale (DASS). Data were analyzed using Structural Equation Model (SEM) techniques with stratification by parent-and-child gender match.

**Results:** One thousand ninety-eight (*n* = 1,098) parent-and-child dyads were recruited, and useful information was obtained. The mean IAT score was 28.6 (*SD* = 9.9) for parents and 41.7 (*SD* = 12.4) for adolescents. Results of the SEM suggested that the effect of parental depression on adolescent IA was mediated through adolescent mental health mainly through adolescent stress (regression weight = 0.33, *p* < 0.001) and less so through adolescent depression (regression weight = 0.19, *p* < 0.001) or parental IA (regression weight = 0.13, *p* < 0.001). Further analysis revealed that these mediating relationships are more significantly manifested in the father-and-son and mother-and-daughter dyads.

**Conclusions:** Result suggested that the relationship between parental mental health and adolescent IA is complex and that adolescent mental health and parental IA also play important roles as mediating factors. These results have direct implications on the treatment and prevention of IA among young people.

## Introduction

Internet addiction (IA) is a disorder that is characterized by compulsive use of the Internet and associated withdrawal and tolerance (1). The craving for Internet and loss of control of Internet usage lead to poor time management and social problems (2). Familial and parental factors of adolescence IA have been drawing some attention, and there has been a growing effort in this particular area of research (3–20). Many different possible factors have been studied, including family conflict or cohesion and satisfaction (3, 4, 6, 9, 12, 17, 20), parenting styles, supervision, or monitoring (5, 7, 10, 11, 15, 19, 20), family communication (4, 8, 13), parental attitudes toward excessive Internet use (3, 4), and parental drinking (3, 6, 14). A recent study reviewing most of these familial factors suggested that adolescent IA was significantly associated with divorced parents, single parent household, and being the only child in the family (21). While the review study only focused on IA among Chinese youth who may not present any problems, all these studies suffered from the same drawback with parental information collected through the report of the child, not from the parents *per se* (21).

Mental health in adolescents, such as attention deficit and hyperactivity disorder, obsessive–compulsive disorder, depression, anxiety, hostility, alcohol abuse, and sleep disturbance, have been reported as comorbidities of IA (7, 22–25). However, in the area of familial and parental factors as potential risk or protective factors of IA among young people, the relationship between parental mental health and adolescence IA has not been studied before 2014. To bridge this gap of knowledge and to address some of the methodological weaknesses in many of the previously reported studies, the author conducted a parent-and-child dyad study with a specific aim to explore the association between parental mental health and their children's IA (26). Results from this study suggested a significant relationship between parental mental health and adolescence IA. Adolescents in the moderate to severe IA group were three times [odds ratio (OR) = 3.03, 95% CI = 1.67–5.48] as likely to have parents classified with moderate to severe depression when compared to those in the mild or normal group after adjusting for potential confounding factors, including the mental health of the child (26). However, no significant relationship between parental anxiety, as well as stress, and adolescence IA was found. While considering the effect of parental behaviors on the behaviors of their children, parental Internet use may also be an influential parental factor on the Internet usage of their children. The author further explored the relationship between parental IA and the IA of their children, taking into consideration adolescent mental health (27). The results also suggested a significant parent-and-adolescent IA relationship in the group of adolescents with a low stress level (*OR* = 3.18, 95% CI = 1.65–6.14), but not in the high stress group.

Integrating the results from these studies suggests that the relationship between parental mental health and their children's IA is complex and other factors, such as parental IA and mental health status of the child, are also involved. Further theorization is necessary for a possible mechanism through which the relationship between parental mental health and adolescent IA can be understood. One possible mechanism is that these factors may be involved in a mediating rather than an interactive manner in the relationship. This hypothesis is implicated by the results of the two previous studies (26, 27). Furthermore, the significant association between parent-and-child mental health statuses has been established and that different genders of the offspring may have different levels of susceptibility to their parent's problems depending on the gender of the parents (28, 29). Hence, the gender of the parent and child may also play an important role in attenuating the aforementioned complex relationships, especially in behavioral modeling.

In view of these considerations, the aim of this study is to further examine the relationship between parental mental health and their children's IA, taking into consideration parental IA, the mental health of children, as well as the genders of the parent and child. Given the results obtained in the previous studies, parental depression will be the main focus in this study.

## Materials and Methods

The study was conducted in March 2014 utilizing a cross-sectional parent-and-child dyad design. The sample populations were high school students aged 13–17 years and their parents. The total student population of adolescents attending high schools during the study period within a specific local school region was included in the sample frame, from which the sample was generated. By regulation, all high schools are registered with the Hong Kong Education Bureau, the governmental body responsible for the education of all pre-tertiary students in Hong Kong. From the list of registered high schools, two schools within a local school region were randomly selected to be the target schools. In each school, a class was then randomly selected from each grade, from grades 7 to 11, with all students and their parents in the class invited to participate in the study. Ethics approval for the study was granted by the Hong Kong University of Education (formerly Hong Kong Institute of Education).

With the endorsement of the school principals, students, and one of their parents (if students lived with both parents) were invited to participate in the study. Since all students provided their family details upon enrollment to the school, thus their parents/stepparents were identified readily. School principals and teachers provided the students and parents with the study information, consent form, and survey questionnaire. After studying the information, students and parents were invited to sign the consent form before filling in the Student's or Parent's Health Survey Questionnaires, respectively. Should the student lived with both parents, the student would nominate a parent and seek his/her consent to participate and to fill in the questionnaire. Completed questionnaires were returned to the school in a sealed envelope to safeguard the confidentiality of the respondents.

The Health Survey Questionnaires for adolescents and parents included similar questions, with some specifically designed for parents or students. In terms of the definition of parents, it could include both biological and non-biological parents as far as they were living with their children. The sample only included students living with their parents, and none were staying with other relatives, such as grandparents, uncles, or aunts. For the main measures of this study, two major sets of variables were included. They were the IA of adolescents and parents and the mental health of adolescents and parents.

For the measure of IA, the Internet Addiction Test (IAT) designed and developed by Young was used for assessing the risk of addictive Internet use for both adolescents and parents (30). Based on the concepts and behaviors exhibited by pathological gamblers as defined by the Diagnostic and Statistical Manual of Mental Disorders, Fourth Edition (DSM-IV), diagnostic criteria, the IAT was designed as a 20-item self-reported scale. Questions included in the scale specifically reflect typical behaviors of addiction relating to Internet use. An example question is: “How often do you feel depressed, moody, or nervous when you are off-line, which goes away once you are back on-line?” Respondents were asked to indicate the propensity of their responses on a Likert scale ranging from 1 (rarely) to 5 (always). The IAT was validated with good reliability of Cronbach's alpha values ranging from 0.54 to 0.82 for various factors (31). A total score could be calculated, with possible scores ranging from a minimum of 20 to a maximum of 100. In this study, the total raw scores of both adolescent and parent were used in the analyses. In this study, the Chinese version of the IAT, which was translated and validated by Lai et al. (32) was used. Study suggested that translated demonstrated good psychometric properties with high internal consistency and validity (32).

To assess the mental health status of both adolescent and parent, the Depression, Anxiety, Stress Scale (DASS) was used (33). As a fully validated and commonly used instrument, the DASS was designed for the assessment of stress, depressive symptoms, and anxiety with good psychometric properties including strong reliability and validity (33). The authors of the scale emphasize the fact that as the DASS had been designed as a quantitative measure of distress along three axes, it was not meant to be a categorical assessment of clinical diagnosis (33). Nevertheless, it could be used for identifying individuals who were of high risk of mental health problems with high scores in the subscales indicating a greater likelihood of depression, anxiety, or stress. As the validity of DASS among adults has been demonstrated, it has also been recommended for use among children and adolescents (33, 34). In this study, the total raw scores of each subscale were used in the analyses. Owing to the results obtained in a previous study by the author, which suggested that parental depression was the only mental health variable significantly associated with adolescent's IA, parental anxiety and parental stress were not included in the analyses as part of the parental mental health in this current study. The Chinese version of the DASS was also provided by the original authors of the scale (35).

Other information collected in the surveys included the age and sex of the adolescents and parents, demographics, location of family residence, some details on the means and patterns of accessing the Internet for both adolescents and parents, as well as parental occupation. In this study, the sexes of both adolescent and parent were used to generate the parent-and-child gender matching groups resulting in four, namely, Male–Male, Male–Female, Female–Male, and Female–Female groups. Separate analyses were conducted for the overall dataset and each of these four matching groups.

Data were analyzed using the IBM SPSS V23.0 and AMOS V23.0 statistical software programs. Using SPSS 23.0, descriptive analyses were conducted using percentages, means, and standard deviation. The focus of the study was to further examine the relationship between parental mental health, particularly, depression, and adolescence IA, taking into consideration the adolescent's mental health and parental IA. Of specific interest was to test the hypothesis that the effect of parental mental health on adolescence IA was mediated by adolescent mental health and parental IA. To elucidate these relationships, the Structural Equation Model (SEM) approach with maximum likelihood methods was applied to the data for testing various path models using the AMOS 23.0 statistical software. The goodness of fit of different models to the data was examined using multiple criteria. These included the reduced chi-squared statistics (χ^2^/*df* ), Comparative Fit Index (CFI), root mean-square error of approximation (RMSEA), and the Akaike Information Index (AIC) with a χ^2^/*df* <5, CFI >0.90, RMSEA <0.05, and a lower AIC indicating a better fitted model (36). For the examination of various possible models, the following model fitting procedures were adopted. First, an initial model with parental mental health as the study variable and adolescence IA as the outcome variable, with all adolescent mental health variables including depression, anxiety, and stress, as well as the parental IA as mediating variables, was fitted to the data as a single group. Second, after examining the results obtained on the model fit statistics of the initial model, the model was modified based on the empirical evidence before refitting to the data again as a single group. Third, the refitted model was then examined again based on the model fit statistics for its acceptability. Fourth, once an acceptable model was derived, the same model was then fitted to subsets of the data in accordance to the four gender matching groups for model fit analysis.

## Results

One thousand ninety-eight (*n* = 1,098) parent-and-child dyads responded to the survey providing usable information and allowed matching of parent-and-child data. This accounted for 95.3% of the total parents completing the questionnaire. No statistically significant differences were found in the comparisons of demographics, including age, sex, grade, and place of birth between those students with a respondent parent and those whose parent did not respond. The parent-and-child characteristics of the respondents were summarized in Table 1. In terms of mental health, on the whole, parents scored lower in all subscales than adolescents with a mean parental depression score of 1.5 (*SD* = 2.6), anxiety score 1.6 (*SD* = 2.6), and stress score 2.5 (*SD* = 3.1) in comparison to 3.6 (*SD* = 3.8), 3.6 (*SD* = 3.4), and 4.9 (*SD* = 4.0) for adolescents, respectively. For IA, the mean IAT score was 28.6 (*SD* = 9.9) for parents and 41.7 (*SD* = 12.4) for adolescents.

**Table 1:** Frequency(%) or mean (SD) of the characteristics of child-and-parent dyad (*n* = 1,098).

**Characteristics of respondents**	**Frequency (%) or Mean (SD)**
**CHILD CHARACTERISTICS**	
Sex	
Male	483 (44.0)
Female	614 (56.0)
Age group	
15 years or older	620 (56.5)
<15 years	478 (43.5)
Born locally	
Yes	767 (69.9)
No	331 (30.1)
Days accessing the internet in the past week	
Everyday	764 (69.8)
4–6 days	129 (11.8)
3 days or less	202 (18.4)
Main device used to access the internet	
Computer	508 (46.3)
Mobile	501 (45.7)
Others	88 (8.0)
Average time spending on the internet	
3 h or more/day	409 (37.9)
<3 h/day	607 (62.1)
Depression	3.6 (3.8)
Anxiety	3.6 (3.4)
Stress	4.9 (4.0)
Internet Addiction score	41.7 (12.4)
**PARENT'S CHARACTERISTICS**	
Sex	
Male	279 (25.5)
Female	814 (74.5)
Age group	
45 years or older	602 (56.5)
<45 years	464 (43.5)
Born locally	
Yes	455 (41.5)
No	641 (58.5)
Occupation	
Professional	160 (14.6)
Semi/Non-professional	157 (14.4)
Others	776 (71.0)
Depression	1.5 (2.6)
Anxiety	1.6 (2.6)
Stress	2.5 (3.1)
Internet Addiction score	28.6 (9.9)

Following the aforementioned procedures of model fitting, the initial model with parental depression as the study variable and adolescence IA the outcome variable, including all adolescent mental health variables with parental IA as mediating variables, was fitted to the data as a single group. Results obtained on the model fit statistics suggest that the model with all three adolescent mental health measures, and parental IA as mediating variable, failed to provide a satisfactory model fit. As a result, the model was modified with the removal of adolescent anxiety as one of the mediating variables. This variable was removed as adolescent anxiety yielded the lowest regression weight in the paths between parental depression and adolescent anxiety, and adolescent anxiety and adolescent IA, when compared to all other paths in the model.

After the removal of adolescent anxiety, the model was refitted to the data for a further examination. Model fit statistics obtained from this model (Model 1) suggested a satisfactory fit of the model to the data as a whole group with all regression weights being significant and the model fit statistic satisfying all criteria [$\chi_{\left(2\right)}^{2}$ = 4.76, *p* = 0.092; χ^2^/*df* = 2.381; CFI = 0.998; RMSEA = 0.035; AIC = 40.672] (Table 2).

**Table 2:** Goodness-of-fit statistics obtained for different model fits.

**Models**	**Model significance**	**χ^**2**^/*df***	**CFI**	**RMSEA**	**AIC**	**AIC of independent model**
Model 1	$\chi_{\left(2\right)}^{2}$ = 4.76, *p* = 0.092	2.381	0.998	0.035	40.762	1547.908
Model 2	$\chi_{\left(2\right)}^{2}$ = 2.44, *p* = 0.296	1.219	0.990	0.041	38.437	267.214
Model 3	$\chi_{\left(2\right)}^{2}$ = 2.88, *p* = 0.237	1.440	0.998	0.036	38.880	490.900
Model 4	$\chi_{\left(2\right)}^{2}$ = 2.06, *p* = 0.357	1.029	1.000	0.014	38.057	185.222
Model 5	$\chi_{\left(2\right)}^{2}$ = 1.84, *p =* 0.399	0.918	1.000	0.001	37.837	640.734

**Figure 1:**
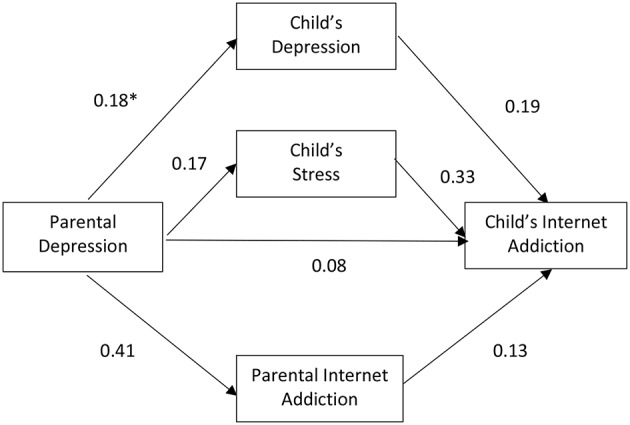
Relationship among parental depression, parental Internet addiction (IA), child's depression, child's stress, and child's IA with all parents and children (Model 1). *Standardized regression weight.

Figure 1 depicts the path diagram of Model 1 with the standardized regression weight of each path. As shown, after taking into consideration adolescent depression and stress, as well as parental IA as mediating variables, the direct effect of parental depression on adolescence IA became minimal. The results suggested that, as a whole group, the effect of parental depression on adolescent IA was mediated through adolescent mental health, mainly through adolescent stress (regression weight = 0.33, *p* < 0.001) and less so through adolescent depression (regression weight = 0.19, *p* < 0.001) or parental IA (regression weight = 0.13, *p* < 0.001).

**Figure 2:**
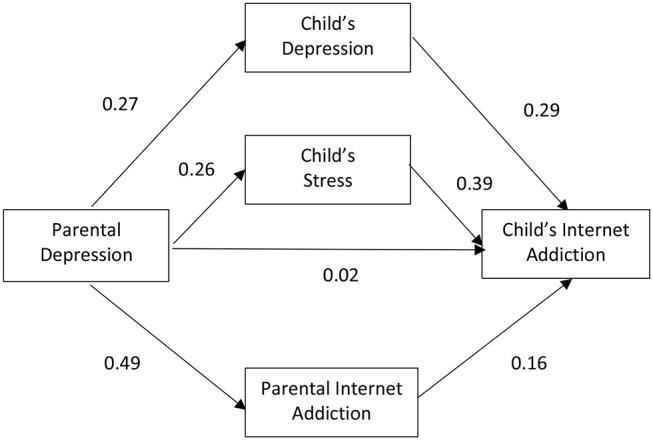
Relationship among parental depression, parental Internet addiction (IA), child's depression, child's stress, and child's IA with male parents and male children (*n* = 234) (Model 2).

**Figure 3:**
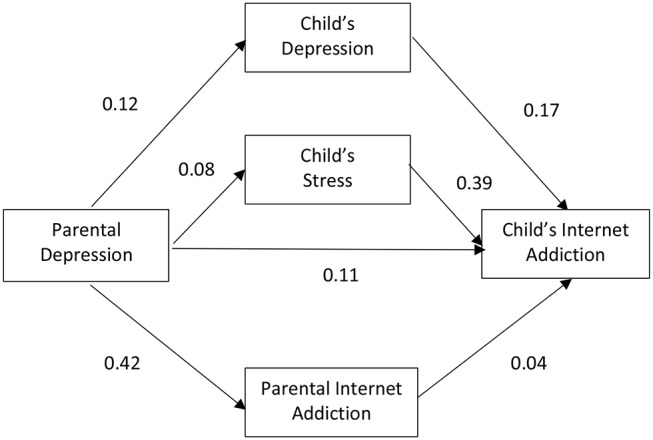
Relationship among parental depression, parental Internet addiction (IA), child's depression, child's stress, and child's IA with male parents and female children (*n* = 145) (Model 3).

**Figure 4:**
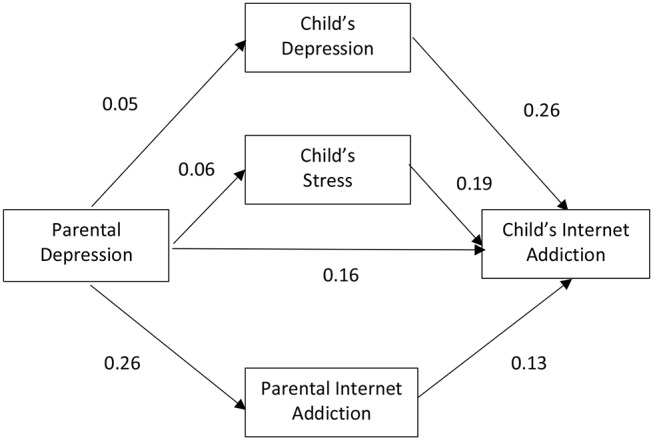
Relationship among parental depression, parental Internet addiction (IA), child's depression, child's stress, and child's IA with female parents and male children (*n* = 247) (Model 4).

**Figure 5:**
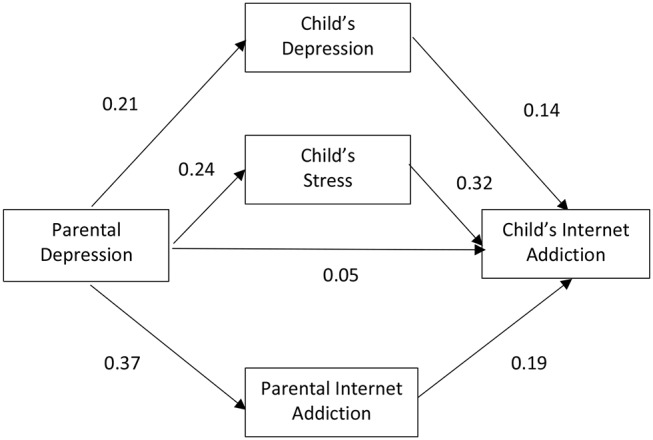
Relationship among parental depression, parental Internet addiction (IA), child's depression, child's stress, and child's IA with female parents and female children (*n* = 466) (Model 5).

Subgroup analyses were conducted after the model for the whole group was deemed satisfactory (Model 1). The model was then fitted to subsets of data in accordance to the parent-and-child matching groups. Figures 2–5 depicts the path diagram of various models with standardized regression weights with the model fit statistic of each model presented in Table 2. According to results obtained on the model fit, all models fitted well to the data with all criteria satisfied. Some models, such as Models 4 and 5, demonstrated a better fit than the others.

Closer inspection on the regression weights of each parent-and-child gender matching models revealed that two of the four models demonstrated a stronger mediating effect through adolescent mental health and parental IA than a direct effect of parental mental health on adolescence IA. These included Model 2 and Model 5, which represent the parent-and-child same sex match groups.

## Discussion

Based on the previous studies, this study further investigates the relationship between parental mental health and adolescent IA paying particular attention to the roles of parent-and-child mental health and parental IA in the relationship. Of further interest is the effect of parent-and-child gender match on this complex relationship. The results obtained indicate that the association between parental mental health, particularly depression, and the adolescent IA is mainly mediated by the mental health status of the child, as well as their own IA. These complex mediating relationships are more significantly manifested in the father-and-son and mother-and-daughter dyads. This is a rather new area of research even within the recently developed discipline of IA or Problematic Internet Use among adults and young people. As reported by the author in previous studies, no reports have been found in the literature on the topic of parental mental health and adolescent IA; the findings of this study are novel and unique.

The results of this study have shed some light on the conceptualization and theorization of the possible mechanism of adolescent IA. The associations between parental mental health, particularly depression, parental IA, and adolescent IA have been identified in previous studies. In this study, further explorations of these relationships reveal that the association between parental mental health and adolescent IA is probably mediated through the mental health problems of the adolescents. These results could be interpreted in light of the Stress, Appraisal, and Coping Theory proposed by Lazarus and Folkman (37). The Stress, Appraisal, and Coping Theory stipulates that, as a response to a stressor or any stressful situations, there evoke a cognitive appraisal process in the individual who is under stress. This process consists of mainly two stages, namely, the primary and secondary appraisals. Primary appraisal refers to an evaluation of whether the stressor will pose a threat and is harmful or simply just a challenge to the individual. On the completion of the primary appraisal, the secondary appraisal follows with an assessment of whether the individual has enough internal or external resources to handle or cope with the stressor effectively (37). The result of the appraisal, incorporating with the usual way of coping of the individual, will determine whether the stressor can be successfully handled. A positive appraisal coupling with an effective coping strategy will result in a positive outcome and vice versa for a negative appraisal marrying with an ineffective coping strategy. In facing family problems, such as parental mental health issue, there triggers a stress reaction in adolescents in a form of distress, depression, and stress. While appraising that the mental health issues of the parent may not be a threat, adolescents may not have the knowledge, understanding, and skills to handle their own distress and the depressed mood. With easy access to the Internet through all sorts of mobile devices at any time, the cyber world becomes an ever ready “safe refuge” for young people to escape to in order to alleviate their distress (38). This could also apply to the parents as suggested by the results that parental depression is also strongly associated with IA in parents. Furthermore, the parental Internet behavior could also become a model of a way of coping for their children such that adolescents adopt it as an acceptable behavior when facing stressful situations. The results obtained on the gender match groups, by and large, echo the phenomena observed in other areas of research (28, 29). This could be understood that, while looking for a role model as part of their natural psychosocial development during that period of growth, adolescents usually adopt the parent of their same sex to be the example and thus they tend to be affected in a greater extent by a parent of the opposite sex.

In countries where there is a strong cultural milieu for parental influence on their children, particularly during the adolescence period, the results obtained from this study have a direct implication on the clinical treatment and prevention of IA among young people. IA in adolescents is not just a problem of young people, but a problem of the whole family. The behavior is likely a reflection and manifestation of familial problems that exert an influence on the mental health of the parents and in turn affect the mental health of their children resulting in the Internet problems. In handling the IA of adolescent, a family therapy approach with special attention paid to the same-sex parent-and-child dyad should be considered. It is also important to assess the Internet behavior of parents as part of the treatment regime in order to determine whether there is any excessive use of the Internet in parents as a way of coping with their own problems. For prevention, parents need to be informed and educated that their own mental health and Internet behavior would exert a significant effect on their offspring. Particularly, psychoeducation in the developmental needs and characteristics of adolescents should be provided to parents that they may unknowingly exert a greater influence on their same-sex children than the other children.

Some strengths and weaknesses have been identified in this study. The study recruits a population-based random sample of adolescents and utilizes standardized and validated instruments for the assessment of all main variables, minimizing some measurement biases. Moreover, a dyad study design allows the collection of information directly from parents and adolescents, thus reducing information bias. In terms of weaknesses, first, information is obtained via a self-reported questionnaire, thus may constitute a report bias in the assessment of variables, for example, both parents and adolescents might have rated themselves more or less severe on depression or IA, although it would most likely be a non-differential bias. Second, since parents self-nominated to participate in the study, there could be a chance that parents with less problems would more likely to self-nominate as participants. This could possibly constitute a confounding situation. Should that be the case, there could be a bias toward the null resulting in a smaller estimated effect but in fact the actual effect should be larger. Third, by nature, this is a cross-sectional study, and the evidence provided from such a study can only be considered as associative and is insufficient to draw any causal inference. This study can be considered as a further exploratory study into the complex relationship between parental mental health and adolescent IA. Future studies of longitudinal nature could be conducted to further examine the possible causal mechanism of the parental influence on their offspring's Internet behavior.

## Data Availability Statement

The datasets generated for this study are available on request to the corresponding author.

## Ethics Statement

The studies involving human participants were reviewed and approved by Human Ethics Committee of the Education University Hong Kong. Written informed consent to participate in this study was provided by the participants' legal guardian/next of kin.

## Author's Note

Part of the results were reported as an abstract and was published in the Proceeding of the 17th International Mental Health Conference on August 10–12, 2016, organized by the Australian and New Zealand Mental Health Association. A similar report was also published as another abstract in the Proceeding of the 3rd Australian and New Zealand Addiction Conference on February 20, 2017, organized by the Australian and New Zealand Mental Health Association. Permission to use these materials has been granted by the Australian and New Zealand Mental Health Association on February 11, 2020.

## Author Contributions

LL was the principal investigator who formulated the research question, developed the study protocol, obtained institutional ethics approval, designed and piloted the survey questionnaire, conducted data analyses, and wrote the manuscript.

## Conflict of Interest

The author declares that the research was conducted in the absence of any commercial or financial relationships that could be construed as a potential conflict of interest.
